# RELATIVE EFFECTIVENESS IN BREAST CANCER TREATMENT: A HEALTH PRODUCTION APPROACH

**DOI:** 10.1017/S0266462315000720

**Published:** 2015

**Authors:** Ruth Puig-Peiro, Anne Mason, Jorge Mestre-Ferrandiz, Adrian Towse, Clare McGrath, Bengt Jonsson

**Affiliations:** Servei Català de la Salut (CatSalut)Ruth.puig@catsalut.cat; University of York; Office of Health Economics; Office of Health Economics; AstraZeneca; Stockholm School of Economics

**Keywords:** Relative effectiveness, Breast neoplasms, Drug evaluation, European Union

## Abstract

**Background:** Pharmaceuticals’ relative effectiveness has come to the fore in the policy arena, reflecting the need to understand how relative efficacy (what can work) translates into added benefit in routine clinical use (what does work). European payers and licensing authorities assess value for money and post-launch benefit–risk profiles, and efforts to standardize assessments of relative effectiveness across the European Union (EU) are under way. However, the ways that relative effectiveness differs across EU healthcare settings are poorly understood.

**Methods:** To understand which factors influence differences in relative effectiveness, we developed an analytical framework that treats the healthcare system as a health production function. Using evidence on breast cancer from England, Spain, and Sweden as a case study, we investigated the reasons why the relative effectiveness of a new drug might vary across healthcare systems. Evidence was identified from a literature review and national clinical guidance.

**Results:** The review included thirteen international studies and thirty country-specific studies. Cross-country differences in population age structure, deprivation, and educational attainment were consistently associated with variation in outcomes. Screening intensity appeared to drive differences in survival, although the impact on mortality was unclear.

**Conclusions:** The way efficacy translates into relative effectiveness across health systems is likely to be influenced by a range of complex and interrelated factors. These factors could inform government and payer policy decisions on ways to optimize relative effectiveness, and help increase understanding of the potential transferability of data on relative effectiveness from one health system to another.

Relative effectiveness can be defined as “the extent to which an intervention does more good than harm compared with one or more alternative interventions under the usual circumstances of healthcare practice” (1). This contrasts with relative efficacy, which is a comparison “under ideal circumstances,” which is usually associated with controlled clinical trials (2). “Comparative effectiveness” is closely related to relative effectiveness (3).

Towse et al. (3) propose an analytical framework, which draws upon production function theory, that describes how certain sets of inputs and processes yield specified outcomes. The aim is to systematically identify and quantify the potential determinants of relative effectiveness. This study reports a first assessment of the framework to help understand the contextual differences between countries that could be associated with differences in effectiveness and relative effectiveness. In recognition of ongoing efforts to develop European Union (EU) -level approaches to assessment, our case study focuses on breast cancer in three countries in the EU.

## OBJECTIVE

To highlight potential cross-country differences in the relative effectiveness of a new drug we reviewed studies investigating reasons for differences in health outcomes in breast cancer. We also reviewed relevant national clinical guidelines and health technology assessment (HTA) reports to understand similarities and differences in the management of breast cancer. We show how our analytical framework can help to understand the factors that might drive differences in relative effectiveness across different settings.

## METHODS

In a separate study in this issue (3), we set out an analytical framework that uses a health production function approach, with health as the output of interest (4). Inputs (“factors” or “determinants”) are classified according to the level at which they operate: patient level (i.e., individuals’ clinical or socio-demographic characteristics); provider level; and the level of the healthcare environment or system. The relative effectiveness of a drug is the additional net output (health) achieved by adding a new drug to usual care or substituting it for another treatment. In this study, we use breast cancer as a case study to identify evidence on the factors associated with health outcomes, drawing on findings from England, Spain, and Sweden.

In selecting a disease area for our case study, we considered several potential tracer conditions including cardiovascular disease, Alzheimer's disease, schizophrenia, cancer, osteoporosis, and rheumatoid arthritis. We selected breast cancer because it is a common condition, is a high clinical priority in all three countries, and new drugs enter the market regularly. Outcomes are driven by both drug and nondrug interventions, as well as by the coordination of care across different settings, and the care pathway covers prevention, early detection, diagnosis, surgery, and adjuvant therapy.

The selection of countries was mainly driven by the likelihood that data would be available for most of the factors we wanted to investigate. We, therefore, decided to limit our choice to countries with similar gross domestic product (high income countries), that had good data on usage and cost, and that varied in technology diffusion and health outcomes. Pragmatically, national clinical guidelines would be accessible only if published in English, Spanish, or Swedish, and this factor helped us to finalize our selection. Our three study countries, England, Spain, and Sweden, have published clinical guidelines on breast cancer, which provide an indication of national priorities and inputs that may influence outcomes. Two of the three countries (England and Sweden) have also assessed the cost-effectiveness of (some) breast cancer drugs.

To identify the data that would be needed to populate a health production model for breast cancer, we undertook a review of the literature. We also reviewed national clinical guidelines and HTA reports.

### Literature Review

A recent review of studies explored the extent of any variation in relative efficacy and relative effectiveness of medicines used in one or more EU Member States (5). The review found little empirical evidence on cross-country differences, and no cross-border observational studies to compare effectiveness in routine practice. For the purpose of this article, we, therefore, simplified our approach: focusing on breast cancer, we searched for studies that investigated determinants of health outcomes such as mortality or quality of life in one or more of our countries of interest (Sweden, Spain, the United Kingdom). We included regression analyses and registry studies published between January 2000 and August 2011. The search strategy was designed for Medline based on key search terms agreed by three of the authors (Table 1) and then adapted to run on EMBASE. The Medline strategy is available in online Supplementary Table 1.

**Table 1.** Terms Used in the Electronic Search Strategy

Term category	Examples
Illness terms	Breast cancer/neoplasm/ carcinoma
Health outcomes terms	Mortality/ survival/death rates, quality of life, health related quality of life (HRQL) and life expectancy
Setting terms	England, Spain and Sweden or cross-country, international, comparison, benchmarking
Generic terms for factors	Cause, driver, explanatory, covariate, determinant, etc.
Study design	Time trends/series analysis, regression/survival/logistic models multivariate/bivariate/univariate analysis.

*Note*. Both interventional (experimental) and observational studies were eligible for inclusion. The search strategy used for Medline is available online (Supplementary file 1).

**Figure 1.**
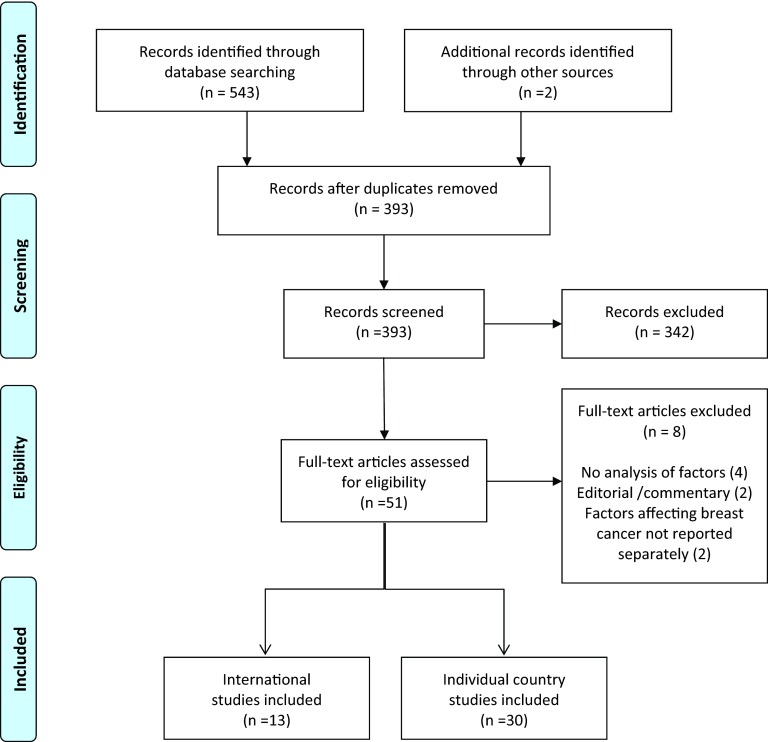
Flow diagram showing the selection process for studies included in the literature review.

Titles and abstracts from the searches were screened for eligibility by two of the review team (R.P.P., A.M.). To be eligible for inclusion, studies needed to explicitly investigate determinants driving differences in outcomes, either across countries (international comparative studies) or within countries (individual country studies). Potentially eligible studies were identified by two authors (R.P.P., A.M.) and assessed for inclusion by one author (RPP). Figure 1 shows the study selection process. One member of the review team (R.P.P.) extracted the data from each study into a template, providing details of the study design, countries covered by the study, data sources, health outcomes and findings (see online Supplementary Tables 1 and 2). As shown in Table 2, the factors identified were then grouped into the framework categories reflecting the level of influence (individual, provider, and national level) using the template from Table 1 in Towse et al. 2015 (3). These data were checked by a second reviewer (A.M.).

**Table 2.** Factors Affecting Breast Cancer Outcomes: Findings from the Review

Influence level	Category	Key findings	Study references
Individual / patient level factor	Demographic characteristics	**Age**: Older patients tend to have worse prognosis and therefore lower survival rates. This is partly due to greater co-morbidity, but the observed large differences in the intensity of treatment of older patients cannot be explained by co-morbidity alone.	(6; 13; 15; 30; 33)
		**Lifestyle**: Smoking, diet and other lifestyle characteristics (number of children, age at first pregnancy, age at menarche and menopause, etc.) are different across populations.	(8; 10; 11; 17; 44)
		**Socio-economic status**: Women with higher socio-economic status (i.e. with higher income, more skilled work and a high level of education) have a statistically significantly higher survival rate, even after adjustment for tumor size and age at diagnosis.	(9; 19; 21; 27; 35; 36; 39; 40; 42; 46)
	Clinical characteristics	**Stage at diagnosis** is an important predictor of differences in 5-year relative survival rates across countries (after adjusting for age, all-cause mortality, number of nodes examined at diagnosis and surgery).	(7; 10; 12–15; 19; 30; 48)
		**Co-morbidity**: Some treatment options (radiotherapy or chemotherapy) may be contraindicated because of specific co-morbidities.	(10; 33)
		**Tumor pathology** affects breast cancer outcomes	(13)
		**Genetics**: Cancer-specific survival in parents predicts survival from the same cancer in their children.	(37; 38; 43)
Provider level factors	Provider characteristics	Differences in **medical practice and treatments** can influence survival rates.	(13; 15; 33)
		**Access to hospitals** Delayed time to diagnosis and long waiting lists negatively impact cancer survival.	(10; 17; 18; 24; 28)
		**Access to ambulatory care**: longer travel time to the GP associated with late stage at diagnosis of breast cancer, after adjusting for age, sex, and deprivation.	(20)
		Introduction of **multidisciplinary teams** associated with improvements in processes of care for breast cancer patients, but not with improved survival.	(6; 23; 25)
		**Data quality / comparability**: there are differences between countries in the methods and specificity of certifying cause of death which partially explains differences in reported breast cancer mortality rates.	(6; 8)
Environment/ health care system level factors	Population health	**Infant and total mortality rates**; **life expectancy** at birth; **unemployment** rate independently and significantly associated with cancer survival.	(6; 15; 16)
		**Awareness** of symptoms may lead to early diagnosis and access treatment improving the health outcomes	(10)
		The distribution of **tumor biology** can differ across populations and lead to differences in cancer outcomes across countries.	(7)
	National /regional guidelines/ regulations	Site-specialist **multidisciplinary teams** introduced as part of a national initiative changed treatment patterns and increased surgical specialization, but the improvement in survival rates was not statistically significant.	(23; 25)
	Service delivery and organisation	**Screening intensity** is associated with increased incidence of early stage breast cancer, which in turn leads to improved overall survival rates (lead time bias, length time bias). Improved mortality rates are less evident.	(6–9; 11; 12; 17; 26; 29; 32; 34; 41; 45; 47; 48)
	Access issues (local/regional/ national)	**Diagnosis and treatment**: Inequalities of access can be partly explained by the national total expenditure on healthcare. Lower use of radiotherapy may adversely affect cancer outcomes. Fewer GPs per thousand population is associated with delays in diagnosis of cancer and worsened prognosis.	(6; 10–13; 15; 17; 18; 28; 29; 31)
		**Hospital equipment / beds**: Number of in-patient beds; number of computerized tomography (CT) scanners per million population positively associated with survival rates.	(16)
	Economy	**Income and expenditure**: Positive association between survival rates and countries’ national income; expenditure on health as a proportion of GNP; and public expenditure on health as percentage of total health expenditure.	(7; 12; 13; 16; 18)
	Environment	**Sunlight**: Patients diagnosed in summer and autumn have a longer survival than patients diagnosed in winter. Vitamin D levels play a relevant role in tumor growth suppression.	(22)

*Note*. Two international studies (8; 14)and one based in Spain (31) discussed the quality and efficacy of care in relation to their findings but none formally tested for it.

### Clinical Guidance Review

To identify similarities and differences in recommended care pathways across our study countries, clinical guidelines for the treatment of breast cancer and relevant HTA reports were reviewed. We searched the Web sites of national HTA agencies (England and Wales, Sweden), Ministries of Health (Spain) and Royal Colleges (Spain), and consulted experts (Sweden). Comparative data on screening programs, and treatment recommendations by stage of disease were extracted and tabulated.

## RESULTS

Forty-three studies were included in the literature review. Thirteen of these forty-three were international comparative studies that covered at least two of the three countries in our case study (6–18). The remaining thirty studies were national, investigating individual countries. Nine studies covered England (19–27), four were set in Spain (28–31), and seventeen were set in Sweden (32–48).

The review of national guidance (either clinical guidelines or HTA reports) identified five documents on breast cancer care for England and Wales (49–53), three from Spain (54–56), and six from Sweden (57–62). The Cancer Strategy document published by the Spanish Ministry of Health (54) makes no treatment-specific recommendations, so we also reviewed the two Spanish Society of Medical Oncology (SEOM) guidelines (55;56) although these are not “official” guidance. In all countries, guidelines covered the whole disease pathway incorporating early, advanced, and metastatic disease.

Table 2 provides an overview of factors affecting breast cancer outcomes identified from the literature review. It groups them according to the multilevel approach: “individual level,” “provider level,” and “environment and healthcare system level” set out in Towse et al. (3) (Table 1). The table lists the studies that either tested for determinants, or commented on them. We discuss the key factors below.

### Individual Level Factors

At the individual level, several demographic factors were consistently associated with poorer outcomes in breast cancer patients, including older age, socio-economic status, and lifestyle factors (smoking status). Older women (aged 75 and over) had lower survival rates than younger women. Although this is partly explained by stage at diagnosis (13)—older women are more likely to present with late stage disease—a Swedish study found that survival differences persisted and were more pronounced in older women with late stage disease than clinically comparable (but younger) women. Older women underwent less intensive diagnostic activity, and less aggressive treatment, even after adjusting for comorbidity (33). Evidence from England and Sweden suggested that women with lower socio-economic status have worse survival, after adjusting for tumor size and age (19;42) and that better educated women are likely to have a better prognosis (39;40). A Swedish study found that smoking status independently increased the risk of death (after adjusting for age and stage of disease) (44). We found no direct evidence on treatment concordance (adherence).

In terms of individuals’ clinical characteristics, there was strong evidence that disease stage at diagnosis is an important—and perhaps *the* most important—predictor of cross-country differences in 5-year survival. However, stage at diagnosis is not, in itself, an “explanation”; rather, it begs the question of why disease stage differs across countries. Possible reasons include screening intensity, access to diagnosis and treatment, and public awareness (which we consider below). Tumor pathology, in particular, the proportion of women with node negative disease, accounts for some differences in survival (13), and Swedish studies found that genetic (familial) determinants also affect prognosis and survival (37;38;43). Women with specific comorbidities may have fewer treatment options, for instance if they are unsuitable for radiotherapy or chemotherapy (33). However, we found no study that explicitly tested the impact of co-morbidity on survival.

### Provider Level Factors

There was less evidence on which features of the healthcare system influence survival, and our searches found no cross-country analyses. Studies from England have investigated the role played by access (travel time) and by multidisciplinary teams (MDTs). Travel time to the GP (general practitioner) was correlated with stage at diagnosis, but there was no consistent relationship between travel time to hospital and survival or stage at diagnosis (20). MDTs improved the process of care but did not significantly improve survival at 1, 3, or 5 years (23;25). However, if average survival for a breast cancer patient is around 7 to 8 years after diagnosis (15), longer follow-up periods may be needed to detect an effect.

Other studies have considered access to diagnostic facilities and to treatments, and waiting times between symptom onset and treatment. The importance of access to diagnostic facilities is well-recognized, and we discuss this in relation to screening programs (see below). An English study analyzed data from the Northern and Yorkshire Cancer Registry and Information Service (NYCRIS) to compare 3-year survival rates for those diagnosed between 1982 and 1990 with cases diagnosed between 1991 and 1999 (24). In all age groups, 3-year survival improved significantly between the two periods. Stage at diagnosis explained all the improvement in those aged over 65, and explained most of the improvement in women aged below 65. Although the uptake of systemic treatment (chemotherapy and hormone treatment) increased substantially over time, systemic treatment had no statistically significant effect in explaining improvements in prognosis in any age group or overall. However, there are several reasons why this “negative” finding for treatment effect needs to be interpreted carefully. First, 3-year survival may be too short a time to robustly assess the impact of systemic therapy on mortality. In addition, data on stage at diagnosis were missing for a large proportion of cases, particularly in the earlier period. This “stage migration” could have led to greater misclassification bias in the first period, which could, therefore, overstate the role of stage in explaining survival improvement. Lastly, the study did not test for an interaction between stage at diagnosis and treatment uptake, so did not isolate the effect of earlier treatment *per se*. Further details of this study (24) are available in online Supplementary Table 1.

Finally, the quality and consistency of data recording is known to vary across countries, and there are differences between countries in the methods and specificity of certifying cause of death (6;8). However, a recent analysis found that even “implausibly extreme” assumptions about data errors could not account for all the observed cross-country differences in survival (18).

### National / Environmental Factors

There are national screening programs in operation in England and Wales (63) and in Sweden (64). In Spain, screening programs are managed and run on a regional basis. Table 3 summarizes the characteristics of the screening programs in terms of the target population and screening interval, based on the review of clinical guidelines.

**Table 3.** Breast cancer screening policy in three European countries

	England (and Wales)	Spain	Sweden
Competency	National (NHSBSP)	Regional	National (but with regional differences)
Start date	1988	1989 (8)	1986
Target population (age)	50–70 • Women aged 40–50 invited for annual mammography if at significant risk • Women >70 years: not invited but can self-refer every three years • eligibility to be extended to 47–73 years by 2016	50–69 (54)	40–74 • All women aged 50–69 • 60–70% of counties also invite women aged 40–49 • 50% counties also invite women aged 70–74 (65)
Screening interval	Every three years	Every two years	18–24 months, depending on age
% estimated uptake in target population (year assessed) (64)	75% (2008)	64% (2006)	80% (2008)

Notes: NHSBSP: National Health Service Breast Screening Programme (http://www.cancerscreening.nhs.uk/breastscreen/)

Sources: Botha 2003 (8); Ministerio de Sanidad y Politica Social 2010 (54); Wilking 2009 (64); Autier 2011 (65)

The intensity of screening activity was strongly associated with improved survival, although evidence for an impact on mortality rates was mixed (6;32). Both national screening programs and opportunistic screening increased the incidence of early stage breast cancer. This improves overall survival rates, reflecting both the effect of earlier treatment and lead time bias. However, countries that have not introduced screening have also seen improvements in survival (6;8), suggesting that other factors play a role.

Evidence on the role of national guidelines was sparse, in terms of both the extent of implementation and the effect on outcomes. Our review of national guidance found few differences in recommendations for treatment of breast cancer, but variation in the date of issue and of the scope of guidance, as well as its implementation, may be important. A Swedish study investigated regional differences in survival, and found that suboptimal diagnostic activity in one county explained the variation. Services were reorganized in this county: multidisciplinary working was better staffed and co-ordinated, screening and diagnostic activity were quality assured, and treatment recommendations were implemented. When guideline adherence improved in these ways, survival also improved (34). An evaluation of the effects of 1995 Calman-Hine report, which introduced national cancer guidelines, found that adherence varied across English regions (23). A study found evidence that care processes had improved as a result of both the Calman-Hine report and the subsequent English Cancer Strategy (2000), but improvements in survival were not statistically significant (25).

Several international studies found that countries with higher national income, and that spent a greater proportion on healthcare, also had better survival rates (7;12;13;16;18). This may be due to improved access to care. For example, countries with higher national income may be able to afford better equipped hospitals; the number of in-patient beds and computerized tomography (CT) scanners per million population were found to be positively associated with survival (16). However, some of this improvement in survival may be an artefact of improved detection methods (e.g., screening programs) which increases the incidence of “over diagnosed” cancers (see Table 1).

## DISCUSSION

Our case study is not a definitive assessment of the validity of our framework, but rather a first attempt to explore how a health production approach can help identify the factors that should be considered in an assessment of the relative effectiveness of a new drug. These factors could potentially be used to optimize effectiveness in routine practice. Engagement from broad group of stakeholders (including providers) would be crucial to the success of this process, and we set out below the types of challenge they would need to resolve.

### Choice of Outcome Measure

Cross-country differences in breast cancer outcomes are well documented (6;7;13;14;64). However, the outcome measure used to assess relative performance across countries can give very different results in terms of ranking. When our three countries are assessed by 5-year survival rates, Sweden is ranked first and the United Kingdom is ranked last (14); but an analysis of mortality trends from 1989 to 2006 ranked Spain first and Sweden last (6). To understand this apparent discrepancy, we need to recognize that survival is a “complex indicator of a country's performance” (7). Longer survival may reflect later death and/or earlier diagnosis—and earlier diagnosis may reflect screening intensity. But earlier diagnosis that does not lead to later death is of questionable benefit to patients. Comparisons based on survival may, therefore, be misleading, if differences in survival do not reflect reductions in mortality. A recent international comparison suggested screening did not play a direct part in reductions in mortality (65). Both survival *and* mortality may need to be considered alongside incidence if valid assessments of prognosis are to be made (15;66).

### Data Limitations

A limitation is that we have only identified factors reported in the literature, and there may be other important drivers that have not been assessed. For example, we found no study that isolated the impact of hormone replacement therapy (HRT) on outcomes. HRT is associated with an increase in the risk of breast cancer (67;68), but only an estimated 3 of 100 breast cancers is related to use of HRT (69). As use of HRT varies and breast cancers induced by HRT may be less aggressive, variations in HRT prescribing across countries are likely to influence international differences in survival rates in a complicated way.

Most of the evidence related to the individual level, which probably reflects data availability—cancer registries include an array of patient characteristics, but comparable information on countries’ healthcare provider systems must be added from external sources. Where access to treatment was assessed, this typically did not take account of dose or duration of treatment. Conversely, we found more evidence on national factors, such as screening programs. Subsequent studies need to further elucidate the factors that may influence breast cancer outcomes, ideally in consultation with clinical experts and possibly drawing on additional (unpublished) data sources such as those documenting differences in resource availability, or spend on breast cancer. They would need to take account of evidence of the impact of genetic variations on both prognosis and choice of therapy.

### Causality or Association?

A further shortcoming of our review is that it reports associations between health outcomes and various factors, but it is less clear whether the relationships are causal. This is because most of our studies are retrospective analyses of observational data. The quality of this type of study is heavily dependent upon the number of observations, the underlying data quality (which is rarely reported in journal articles), the functional form of the model and whether there are confounding factors that are not, perhaps cannot be, taken into account. To explore causality would require different study designs, such as randomized trials. However, these are not feasible when investigating the impact of national factors. Even if associations are robust, they shed little light on drivers relating to the inputs and activities included in the care given, which will impact on how a treatment is used and what, if anything, it displaces. There may also be interactions and correlations between the factors we identified, both within and between different levels, for instance, national income is likely to be correlated with individuals’ educational level and individuals’ stage at diagnosis will be linked to system level screening policy. This problem is perhaps more complex for breast cancer than for some other diseases, such as acute conditions, although most chronic diseases are managed through a combination of screening, diagnosis, lifestyle alterations or interventions, and drug treatment.

## CONCLUSIONS AND POLICY IMPLICATIONS

Based on our review of studies comparing breast cancer outcomes and of guidelines/HTA reports in three European countries, we believe that the way efficacy translates into relative effectiveness across health systems is likely to be influenced by a range of complex and interrelated factors. These comprise not only the genetic and other biological and behavioral patient factors mentioned by Eichler et al. (2) (which we term “individual” patient level factors in our model) but also the characteristics of the providers and healthcare environment and system-level factors. For example, the importance of stage at diagnosis begs the question of why stage of disease differs across countries. Arguably, this finding reflects the conclusion of Eichler et al. (2) that “where there is an apparent large gap between efficacy and effectiveness, one is not looking at a drug problem but at a healthcare delivery problem, and the focus of remedial action should be shifted to improving real life performance.”

Relative effectiveness is a current policy issue in Europe, and this is why our case study is focused here. In principle, the same issues arise in any context where drugs are approved centrally but where there may be significant regional variations in how the drugs are used in practice and, therefore, differences in relative effectiveness. By recognizing that impediments to improving health can arise at several levels, policy makers in any jurisdiction can begin to explore ways to optimize relative effectiveness. Studies that show differences in relative effectiveness between countries, or that identify factors suggesting these exist, provide one way to identify how health system performance can be improved.

Careful consideration of the determinants within our framework may also aid discussions on the extent to which evidence for HTA based decision making can be shared across health systems, and identify the data required for robust comparisons. In some cases, it will be reasonable to expect evidence on relative effectiveness to be transferable; in other cases, it may be possible to anticipate and adjust for expected differences in relative effectiveness between countries, and so use evidence from one country in another. In other cases, however, an understanding of relative effectiveness in a country may generate questions that cannot be answered by existing evidence and that require a bespoke study.

## Supplementary material

For supplementary material accompanying this paper visit http://dx.doi.org/10.1017/S0266462315000720. click here to view supplementary material

## CONFLICTS OF INTEREST

Puig-Peiro, M.Sc. reports grants from Pfizer during the conduct of the study and grants from The Association of the British Pharmaceutical Industry outside the submitted work. At the time of writing the report, Dr. Puig-Peiro was working at the Office of Health Economics. Her new affiliation is the Catalan Health Service and she does not have conflict of interests. Dr. Mason reports grants from Pfizer (contract with OHE Consulting) during the conduct of the study and grants from Novartis (contract with OHE Consulting) outside the submitted work. Dr. Mestre-Ferrandiz reports grants from Pfizer during the conduct of the study and from The Association of the British Pharmaceutical Industry outside the submitted work. Professor Towse reports grants from Pfizer during the conduct of the study and from The Association of the British Pharmaceutical Industry, outside the submitted work. Dr. McGrath reports grants from Pfizer during the conduct of the study and from Pfizer and AstraZeneca outside the submitted work. Professor Jönsson reports personal fees from Pfizer, during the conduct of the study.

## References

[1] High Level Pharmaceutical Forum. Core principles on relative effectiveness. Brussels: European Commission: Healthcare Industries Working Group on Relative Effectiveness; 2008:10.

[2] Eichler H-G, Abadie E, Breckenridge A, et al. Bridging the efficacy-effectiveness gap: A regulator's perspective on addressing variability of drug response. Nat Rev Drug Discov. 2011;10:495–506. 21720406 10.1038/nrd3501

[3] Towse A, Jonsson B, McGrath C, et al. Understanding variations in relative effectiveness: A health production approach. *Int J Technol Assess Health Care*. (submitted). 10.1017/S0266462315000719 PMC4824958 26837803

[4] Jönsson B. Relative effectiveness and the European pharmaceutical market. Eur J Cancer. 2011;12:97–102. 10.1007/s10198-011-0297-z 21267624

[5] Mestre-Ferrandiz J, Puig-Peiró R, Towse A. Researching the relative efficacy and relative effectiveness of medicines across Europe. OHE Consulting Report. London: Office of Health Economics; 2010.

[6] Autier P, Boniol M, La Vecchia C, et al. Disparities in breast cancer mortality trends between 30 European countries: Retrospective trend analysis of WHO mortality database. BMJ. 2010;341:c3620. 20702548 10.1136/bmj.c3620 PMC2920378

[7] Berrino F, De Angelis R, Sant M, et al. Survival for eight major cancers and all cancers combined for European adults diagnosed in 1995–99: Results of the EUROCARE-4 study. Lancet Oncol. 2007;8:773–783. 17714991 10.1016/S1470-2045(07)70245-0

[8] Botha JL, Bray F, Sankila R, Parkin DM. Breast cancer incidence and mortality trends in 16 European countries. Eur J Cancer. 2003;39:1718–1729. 12888367 10.1016/s0959-8049(03)00118-7

[9] Bray F, Sankila R, Ferlay J, Parkin DM. Estimates of cancer incidence and mortality in Europe in 1995. Eur J Cancer. 2002;38:99–166. 11750846 10.1016/s0959-8049(01)00350-1

[10] Coleman MP, Forman D, Bryant H, et al. Cancer survival in Australia, Canada, Denmark, Norway, Sweden, and the UK, 1995–2007 (the International Cancer Benchmarking Partnership): An analysis of population-based cancer registry data. Lancet. 2011;377:127–138. 21183212 10.1016/S0140-6736(10)62231-3 PMC3018568

[11] Karim-Kos HE, de Vries E, Soerjomataram I, et al. Recent trends of cancer in Europe: A combined approach of incidence, survival and mortality for 17 cancer sites since the 1990s. Eur J Cancer. 2008;44:1345–1389. 18280139 10.1016/j.ejca.2007.12.015

[12] Sant M, Aareleid T, Berrino F, et al. EUROCARE-3: Survival of cancer patients diagnosed 1990–94–Results and commentary. Ann Oncol. 2003;14 (Suppl 5):v61–v118. 14684501 10.1093/annonc/mdg754

[13] Sant M, Allemani C, Capocaccia R, et al. Stage at diagnosis is a key explanation of differences in breast cancer survival across Europe. Int J Cancer. 2003;106:416–422. 12845683 10.1002/ijc.11226

[14] Sant M, Allemani C, Santaquilani M, et al. EUROCARE-4. Survival of cancer patients diagnosed in 1995–1999. Results and commentary. Eur J Cancer. 2009;45:931–991. 19171476 10.1016/j.ejca.2008.11.018

[15] Sant M, Capocaccia R, Coleman MP, et al. Cancer survival increases in Europe, but international differences remain wide. Eur J Cancer. 2001;37:1659–1667. 11527693 10.1016/s0959-8049(01)00206-4

[16] Sant M, EUROCARE Working Group. Overview of EUROCARE-2 results on survival of cancer patients diagnosed 1985–1989 In: Berrino F, Capocaccia R, Estève J, et al., eds. Survival of cancer patients in Europe: The EUROCARE-2 Study, vol. 151. Geneva: WHO Press; 1999.

[17] Sant M, Francisci S, Capocaccia R, et al. Time trends of breast cancer survival in Europe in relation to incidence and mortality. Int J Cancer. 2006;119:2417–2422. 16964611 10.1002/ijc.22160

[18] Woods LM, Coleman MP., Lawrence G., et al. Evidence against the proposition that “UK cancer survival statistics are misleading”: Simulation study with National Cancer Registry data. BMJ. 2011;342:2011. 10.1136/bmj.d3399 PMC3111483 21659366

[19] Davies EA, Linklater KM, Coupland VH, et al. Investigation of low 5-year relative survival for breast cancer in a London cancer network. Br J Cancer. 2010;103:1076–1080. 20736945 10.1038/sj.bjc.6605857 PMC2965868

[20] Jones AP, Haynes R, Sauerzapf V, et al. Travel times to health care and survival from cancers in Northern England. Eur J Cancer. 2008;44:269–274. 17888651 10.1016/j.ejca.2007.07.028

[21] Kaffashian F, Godward S, Davies T, et al. Socioeconomic effects on breast cancer survival: Proportion attributable to stage and morphology. Br J Cancer. 2003;89:1693–1696. 14583771 10.1038/sj.bjc.6601339 PMC2394406

[22] Lim H-S, Roychoudhuri R, Peto J, et al. Cancer survival is dependent on season of diagnosis and sunlight exposure. Int J Cancer. 2006;119:1530–1536. 16671100 10.1002/ijc.22052

[23] Morris E, Haward RA, Gilthorpe MS, Craigs C, Forman D. The impact of the Calman-Hine report on the processes and outcomes of care for Yorkshire's breast cancer patients. Ann Oncol. 2008;19:284–291. 17785759 10.1093/annonc/mdm432

[24] Pisani P, Forman D. Declining mortality from breast cancer in Yorkshire, 1983–1998: Extent and causes. Br J Cancer. 2004;90:652–656. 14760380 10.1038/sj.bjc.6601614 PMC2409595

[25] Rachet B, Maringe C, Nur U, et al. Population-based cancer survival trends in England and Wales up to 2007: An assessment of the NHS cancer plan for England. Lancet Oncol. 2009;10:351–369. 19303813 10.1016/S1470-2045(09)70028-2

[26] Robinson D, Bell J, Moller H, Salman A. A 13-year follow-up of patients with breast cancer presenting to a District General Hospital breast unit in southeast England. Breast. 2006;15:173–180. 16081289 10.1016/j.breast.2005.06.002

[27] Sloggett A, Young H, Grundy E. The association of cancer survival with four socioeconomic indicators: A longitudinal study of the older population of England and Wales 1981–2000. BMC Cancer. 2007;7:20. 17254357 10.1186/1471-2407-7-20 PMC1797185

[28] Cabanes A, Vidal E, Perez-Gomez B, et al. Age-specific breast, uterine and ovarian cancer mortality trends in Spain: Changes from 1980 to 2006. Cancer Epidemiol. 2009;33:169–175. 19766076 10.1016/j.canep.2009.08.010

[29] Fernandez E, Gonzalez JR, Borras JM, et al. Recent decline in cancer mortality in Catalonia (Spain). A joinpoint regression analysis. Eur J Cancer. 2001;37:2222–2228. 11677111 10.1016/s0959-8049(01)00279-9

[30] Larrañaga N, Sarasqueta C, Martinez-Camblor P, et al. Female breast cancer in Gipuzkoa: Prognostic factors and survival. Clin Transl Oncol. 2009;11:96–102. 19211375 10.1007/s12094-009-0321-2

[31] Vilaprinyo E, Rue M, Marcos-Gragera R, Martinez-Alonso M. Estimation of age- and stage-specific Catalan breast cancer survival functions using US and Catalan survival data. BMC Cancer. 2009;9:98. 19331670 10.1186/1471-2407-9-98 PMC2679763

[32] Duffy SW, Tabar L, Chen H-H, et al. The impact of organized mammography service screening on breast carcinoma mortality in seven Swedish counties. Cancer. 2002;95:458–469. 12209737 10.1002/cncr.10765

[33] Eaker S, Dickman PW, Bergkvist L, Holmberg L, Uppsala/Orebro Breast Cancer Group. Differences in management of older women influence breast cancer survival: Results from a population-based database in Sweden. PLoS Med. 2006;3:e25. 16409108 10.1371/journal.pmed.0030025 PMC1326256

[34] Eaker S, Dickman PW, Hellstrom V, et al. Regional differences in breast cancer survival despite common guidelines. Cancer Epidemiol Biomarkers Prev. 2005;14:2914–2918. 16365009 10.1158/1055-9965.EPI-05-0317

[35] Eaker S, Halmin M, Bellocco R, et al. Social differences in breast cancer survival in relation to patient management within a National Health Care System (Sweden). Int J Cancer. 2009;124:180–187. 18844231 10.1002/ijc.23875

[36] Halmin M, Bellocco R, Lagerlund M, et al. Long-term inequalities in breast cancer survival–a ten year follow-up study of patients managed within a National Health Care System (Sweden). Acta Oncol. 2008;47:216–224. 18210298 10.1080/02841860701769768

[37] Hartman M, Lindstrom L, Dickman PW, et al. Is breast cancer prognosis inherited? Breast Cancer Res. 2007;9:R39. 17598882 10.1186/bcr1737 PMC1929105

[38] Hemminki K, Ji J, Forsti A, Sundquist J, Lenner P. Survival in breast cancer is familial. Breast Cancer Res Treat. 2008;110:177–182. 17674192 10.1007/s10549-007-9692-7

[39] Hussain SK, Altieri A, Sundquist J, Hemminki K. Influence of education level on breast cancer risk and survival in Sweden between 1990 and 2004. Int J Cancer. 2008;122:165–169. 17708572 10.1002/ijc.23007

[40] Hussain SK, Lenner P, Sundquist J, Hemminki K. Influence of education level on cancer survival in Sweden. Ann Oncol. 2008;19:156–162. 17785761 10.1093/annonc/mdm413

[41] Jonsson H, Nystrom L, Tornberg S, Lenner P. Service screening with mammography of women aged 50–69 years in sweden: Effects on mortality from breast cancer. J Med Screen. 2001;8:152–160. 11678556 10.1136/jms.8.3.152

[42] Lagerlund, Bellocco R, Karlsson P, Tejler G, Lambe M. Socio-economic factors and breast cancer survival–A population-based cohort study (Sweden). Cancer Causes Control. 2005;16:419–430. 15953984 10.1007/s10552-004-6255-7

[43] Lindstrom LS, Hall P, Hartman M, et al. Familial concordance in cancer survival: A Swedish population-based study. Lancet Oncol. 2007;8:1001–1006. 17921068 10.1016/S1470-2045(07)70282-6

[44] Manjer J, Andersson I, Berglund G, et al. Survival of women with breast cancer in relation to smoking. Eur J Cancer. 2000;166:852–858. 10.1080/110241500447227 11097150

[45] Nystrom L, Andersson I, Bjurstam N, et al. Long-term effects of mammography screening: Updated overview of the Swedish randomised trials. Lancet. 2002;359:909–919. 11918907 10.1016/S0140-6736(02)08020-0

[46] Rutqvist LE., Bern A, Stockholm Breast Cancer Study Group. Socioeconomic gradients in clinical stage at presentation and survival among breast cancer patients in the Stockholm area 1977–1997. Int J Cancer. 2006;119:1433–1439. 16596647 10.1002/ijc.21949

[47] Tabar L, Yen M-F, Vitak B, et al. Mammography service screening and mortality in breast cancer patients: 20-year follow-up before and after introduction of screening. Lancet. 2003;361:1405–1410. 12727392 10.1016/S0140-6736(03)13143-1

[48] Warwick J, Tabar L, Vitak B, Duffy SW. Time-dependent effects on survival in breast carcinoma: Results of 20 years of follow-up from the Swedish Two-County Study. Cancer. 2004;100:1331–1336. 15042664 10.1002/cncr.20140

[49] National Institute for Health and Clinical Excellence. Early and locally advanced breast cancer: Diagnosis and treatment (CG80). Updates and replaces technology appraisal guidance 109 (docetaxel), 108 (paclitaxel) and 107 (trastuzumab). London: National Institute for Health and Clinical Excellence; 2009.

[50] National Institute for Health and Clinical Excellence. Advanced breast cancer: Diagnosis and treatment (CG81). Updates and replaces NICE technology appraisal guidance 62 (capecitabine), 54 (vinorelbine) and 30 (taxanes). London: National Institute for Health and Clinical Excellence; 2009.

[51] National Institute for Health and Clinical Excellence. Hormonal therapies for the adjuvant treatment of early oestrogen-receptor-positive breast cancer (TA112). London: National Institute for Health and Clinical Excellence; 2006.

[52] National Institute for Health and Clinical Excellence. Gemcitabine for the treatment of metastatic breast cancer (TA116). London: National Institute for Health and Clinical Excellence; 2007.

[53] National Institute for Health and Clinical Excellence. Bevacizumab in combination with a taxane for the first-line treatment of metastatic breast cancer (TA214). London: National Institute for Health and Clinical Excellence; 2011.

[54] Ministerio de Sanidad y Politica Social. Estrategia en Cancer del Sistema Nacional de Salud (Cancer Strategy). Spain: Ministerio de Sanidad y Politica Social; 2010.

[55] Clinical Guideline Working Group on behalf of the Spanish Society of Medical Oncology (SEOM). SEOM clinical guidelines for the treatment of early breast cancer. Clin Transl Oncol. 2010;12:711–718. 20974561 10.1007/s12094-010-0584-7

[56] Clinical Guideline Working Group on behalf of the Spanish Society of Medical Oncology (SEOM). SEOM clinical guidelines for the treatment of metastatic breast cancer. Clin Transl Oncol. 2010;12:719–723. 20974562 10.1007/s12094-010-0585-6

[57] Engholm G, Ferlay J, Christensen N, et al. NORDCAN–a Nordic tool for cancer information, planning, quality control and research. Acta Oncol. 2010;49:725–736. 20491528 10.3109/02841861003782017

[58] National Board of Health and Welfare (Socialstyrelsen). Cancer i Sverige, Insjuknande och överlevnad utifrån regional och socioekonomisk indelning. Stockholm: Socialstyrelsen; 2011.

[59] National Board of Health and Welfare (Socialstyrelsen). Nationella riktlinjer för bröstcancersjukvård, Medicinskt och hälsoekonomiskt faktadokument. Stockholm: Socialstyrelsen; 2007.

[60] Onkologiskt Centrum. Bröstcancervården i norra regionen.Regionala öppna jämförelser mellan landsting och sjukhus i norra sjukvårdsregionen. Umeå: Onkologiskt Centrum, 2009.

[61] Styrgruppen för det Nationella Bröstcancerregistret. Bröstcancer, Nationell rapport diagnosår 2008. Stockholm: Onkologiskt Centrum; 2010.

[62] Svenska BröstcancerGruppen (Swedish Breast Cancer Group). Nationella riktlinjer för behandling av bröstcancer. Stockholm: SweBCG; 2010.

[63] Richards M. An independent review is under way. BMJ. 2011;343:d6843. 22028483 10.1136/bmj.d6843

[64] Wilking N, Kasteng F. A review of breast cancer and outcomes in 18 countries in Europe, Asia, and Latin America. Comparator Report. Stockholm: Karolinska Institute; 2009.

[65] Autier P, Boniol M, Gavin A, Vatten LJ. Breast cancer mortality in neighbouring European countries with different levels of screening but similar access to treatment: Trend analysis of WHO mortality database. BMJ. 2011;343:d4411. 21798968 10.1136/bmj.d4411 PMC3145837

[66] Autier P, Boniol M. Caution needed for country-specific cancer survival. Lancet. 2011;377:99–101. 21215872 10.1016/S0140-6736(10)62347-1

[67] Bergkvist L, Bixo M, Björkelund C, et al. Hormone Replacement Therapy (HRT): An evidence based review. Report number: 159. Stockholm, Sweden: Swedish Council on Technology Assessment in Health Care; 2002.

[68] Prentice RL. Postmenopausal hormone therapy and the risks of coronary heart disease, breast cancer, and stroke. Semin Reprod Med. 2014;32:419–425. 25321418 10.1055/s-0034-1384624 PMC4212810

[69] Cancer Research UK. In depth information about breast cancer risk and HRT. http://www.cancerresearchuk.org/about-cancer/cancers-in-general/cancer-questions/hrt-and-cancer-risk (accessed January 31, 2015).

