# Supplementary material for: RELATIVE EFFECTIVENESS IN BREAST CANCER TREATMENT: A HEALTH PRODUCTION APPROACH
Source: Int J Technol Assess Health Care. 2015;31(6):371–9. doi: 10.1017/S0266462315000720 (PMC4824961; doi:10.1017/S0266462315000720)
Supplement: Supplementary file 1 [file S0266462315000720sup001.docx]

## Supplementary Table 1: Search Strategy

### MEDLINE(R) STRATEGY, run 12 August 2011

1. Breast cancer.mp.

2. exp Breast Neoplasms/

3. Breast tumo?r.mp.

4. Breast carcinom$.mp.

5. or/1-4

6. mortality.ti,ab.

7. death.ti,ab.

8. survival.ti,ab.

9. quality of life.ti,ab.

10. HRQL.ti,ab.

11. Mortality/

12. Quality of Life/

13. Survival Rate/

14. Life Expectancy/

15. or/6-14

16. Great Britain/

17. (britain or united kingdom or uk or gb).mp.

18. (england not new england).mp.

19. english.ti,ab.

20. Spain/

21. (spain or spanish).mp.

22. Sweden/

23. (sweden or swedish).mp.

24. (international adj2 comparison$).ti,ab.

25. (cross adj countr$).ti,ab.

26. benchmarking.ti,ab.

27. or/16-26

28. cause$.ti,ab.

29. factor$.ti,ab.

30. driver$.ti,ab.

31. covariate$.ti,ab.

32. explanator$.ti,ab.

33. (independent adj variab$).ti,ab.

34. influenc$.ti,ab.

35. determinant$.ti,ab.

36. ((differenc$ or discrepanc$ or variation$) adj3 (mortality or survival or quality of life or HRQL or life expectancy)).ti,ab.

37. or/28-36

38. 5 and 15 and 27 and 37

39. regression$.mp.

40. Registries/

41. (cancer adj2 (registry or registries)).mp.

42. logistic models/

43. survival analysis/

44. ((multivariate or multi-variate) adj2 analys?s).ti,ab.

45. ((univariate or uni-variate) adj2 analys?s).ti,ab.

46. ((bivariate or bi-variate) adj2 analys?s).ti,ab.

47. (time adj trend).ti,ab.

48. (trend adj analys?s).ti,ab.

49. joinpoint.ti,ab.

50. (time adj series).ti,ab.

51. or/39-50

52. 5 and 15 and 27 and 37 and 51

53. limit 52 to yr="2000 -Current"
